# Photoaging Mobile Apps in School-Based Melanoma Prevention: Pilot Study

**DOI:** 10.2196/jmir.8661

**Published:** 2017-09-08

**Authors:** Titus Josef Brinker, Christian Martin Brieske, Christoph Matthias Schaefer, Fabian Buslaff, Martina Gatzka, Maximilian Philip Petri, Wiebke Sondermann, Dirk Schadendorf, Ingo Stoffels, Joachim Klode

**Affiliations:** ^1^ Department of Dermatology, Venereology and Allergology University-Hospital Essen University of Duisburg-Essen Essen Germany; ^2^ Department of Dermatology and National Center for Tumor Diseases (NCT) University Hospital Heidelberg University of Heidelberg Heidelberg Germany; ^3^ German Cancer Consortium (DKTK) Heidelberg Germany; ^4^ West German Cancer Center University Duisburg-Essen Essen Germany; ^5^ Department of Dermatology and Allergic Diseases University of Ulm Ulm Germany

**Keywords:** melanoma, skin cancer, prevention, mobile apps, smartphones, photoaging, schools, secondary schools, adolescents

## Abstract

**Background:**

Around 90% of melanomas are caused by exposure to ultraviolet (UV) radiation and are therefore eminently preventable. Tanning behavior is mostly initiated in early adolescence, often with the belief that it increases attractiveness; the problems related to malignant melanoma and other skin cancers are too far in the future to fathom. Given the substantial amount of time children and adolescents spend in schools, as well as with their mobile phones, addressing melanoma prevention via both of these ways is crucial. However, no school-based intervention using mobile apps has been evaluated to date. We recently released a photoaging mobile app, in which a selfie is altered to predict future appearance dependent on UV protection behavior and skin type.

**Objective:**

In this pilot study, we aimed to use mobile phone technology to improve school-based melanoma prevention and measure its preliminary success in different subgroups of students with regard to their UV protection behavior, Fitzpatrick skin type and age.

**Methods:**

We implemented a free photoaging mobile phone app (Sunface) in 2 German secondary schools via a method called mirroring. We “mirrored” the students’ altered 3-dimensional (3D) selfies reacting to touch on mobile phones or tablets via a projector in front of their whole grade. Using an anonymous questionnaire capturing sociodemographic data as well as risk factors for melanoma we then measured their perceptions of the intervention on a 5-point Likert scale among 205 students of both sexes aged 13-19 years (median 15 years).

**Results:**

We measured more than 60% agreement in both items that measured motivation to reduce UV exposure and only 12.5% disagreement: 126 (63.0%) agreed or strongly agreed that their 3D selfie motivated them to avoid using a tanning bed, and 124 (61.7%) to increase use of sun protection. However, only 25 (12.5%) disagreed with both items. The perceived effect on motivation was increased in participants with Fitzpatrick skin types 1-2 in both tanning bed avoidance (n=74, 71.8% agreement in skin types 1-2 vs n=50, 53.8% agreement in skin types 3-6) and increased use of sun protection (n=70, 68.0% agreement in skin types 1-2 vs n=52, 55.3% agreement in skin types 3-6), and also positively correlated with higher age.

**Conclusions:**

We present a novel way of integrating photoaging in school-based melanoma prevention that affects the students’ peer group, considers the predictors of UV exposure in accordance with the theory of planned behavior, and is particularly effective in changing behavioral predictors in fair-skinned adolescents (Fitzpatrick skin types 1-2). Further research is required to evaluate the intervention’s prospective effects on adolescents of various cultural backgrounds.

## Introduction

Skin cancer is the most common malignancy in fair-skinned populations, with melanoma incidence being the highest in New Zealand and Australia (50 and 48 per 100,000 population, respectively) and projected to increase in European countries such as the United Kingdom (from 17 to 36 per 100,000 population) between 2007-2011 and 2022-2026 [1].

Around 90% of melanomas are caused by ultraviolet (UV) exposure and are therefore eminently preventable [2]. Recent data suggest that especially groups with a low genetic risk benefit from UV protection [3,4] and underline the importance of aggressive prevention strategies for young target groups regarding indoor [5] and outdoor [6] UV exposure.

Unhealthy behavior in regard to UV exposure is mostly initiated in early adolescence [7], often with the idea that a tan increases attractiveness [8-10]; the problems related to malignant melanoma and skin atrophy are too far in the future to fathom. Given the substantial amount of time children and adolescents spend in schools, addressing skin cancer prevention in this setting is crucial and provides a unique opportunity to implement melanoma prevention programs [11]. In an attempt to reduce UV exposure, recent experimental studies designed for young target groups aimed at promoting sunscreen use as an end point [12-15] and others used different UV protection behaviors (including avoiding sunbeds) or behavior scores [9,16-23].

Appearance-based approaches were evaluated as superior to health-based approaches in a school-based randomized trial [12], which underlines the well-understood importance of self-perceived appearance for adolescent self-esteem [24,25].

Photoaging interventions specifically, in which a self-portrait (ie, a selfie) is altered to predict future appearance, indicated effectiveness in various behavioral change settings, including smoking cessation [26-28] or prevention [29-33], weight loss [34], and in recent years also in UV protection interventions [35-38].

We recently introduced the free mobile app Sunface, which takes advantage of the broad availability of smartphones and adolescents’ interest in their appearance by photoaging and 3-dimensional (3D) animation of the users’ selfie based on Fitzpatrick skin type and individual UV protection behavior [39]. Afterward, the app explains the visual results, provides guideline recommendations on sun protection plus the ABCDE rule for melanoma self-detection (assess border irregularity, color variety, diameter, and evolution [40]), and offers sharing options via photo or video (Multimedia Appendix 1).

To integrate this photoaging app for melanoma prevention in the school-based setting and to investigate how it would be perceived by adolescents who are most amenable to appearance-based interventions [24], we tested its effectiveness by the use of the mirroring approach in a pilot study. Mirroring means that the students’ altered 3D selfies on mobile phones or tablets were “mirrored” via a projector in front of the whole grade. This approach was previously introduced by our group in the tobacco prevention setting [30].

## Methods

### Participants

We included a total sample of 205 German secondary school students of both sexes in the age group of 13-19 years in our cross-sectional pilot study (median 15 years, SD 1.36; 111/205, 54.1% male; 93/205, 45.9% female) attending the two most common school types in Germany (grammar school: n=136, 66.4%; general comprehensive school: n=69, 33.6%). Almost all participants (201/205, 98.5%) owned a smartphone.

From a risk profile standpoint, 50.7% (104/205) of the participants had a Fitzpatrick skin type of 1 or 2 [41]; current sunbed use was reported by 5.9% (12/205) [42], and 77.0% (157/205) remembered having at least one sunburn in the past [43].

### Intervention

The mirroring approach was implemented by medical students from the Education Against Tobacco nonprofit organization attending the University of Essen in Germany [31,44]. To increase familiarity with the photoaging app (called Sunface) and students’ participation in the mirroring intervention, students were asked to download the app before our visit, via a letter 1 week in advance. By this means, 36.3% (74/205) had the app on their mobile phones when we visited the schools.

**Figure 1:**
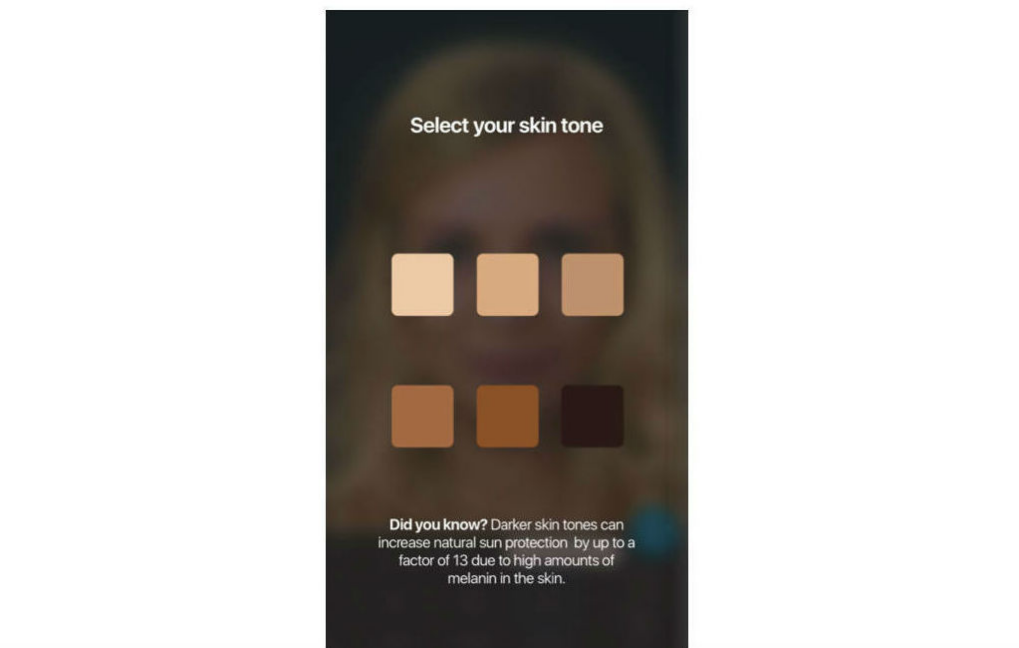
Start of the app: the user picks their Fitzpatrick skin type.

**Figure 2:**
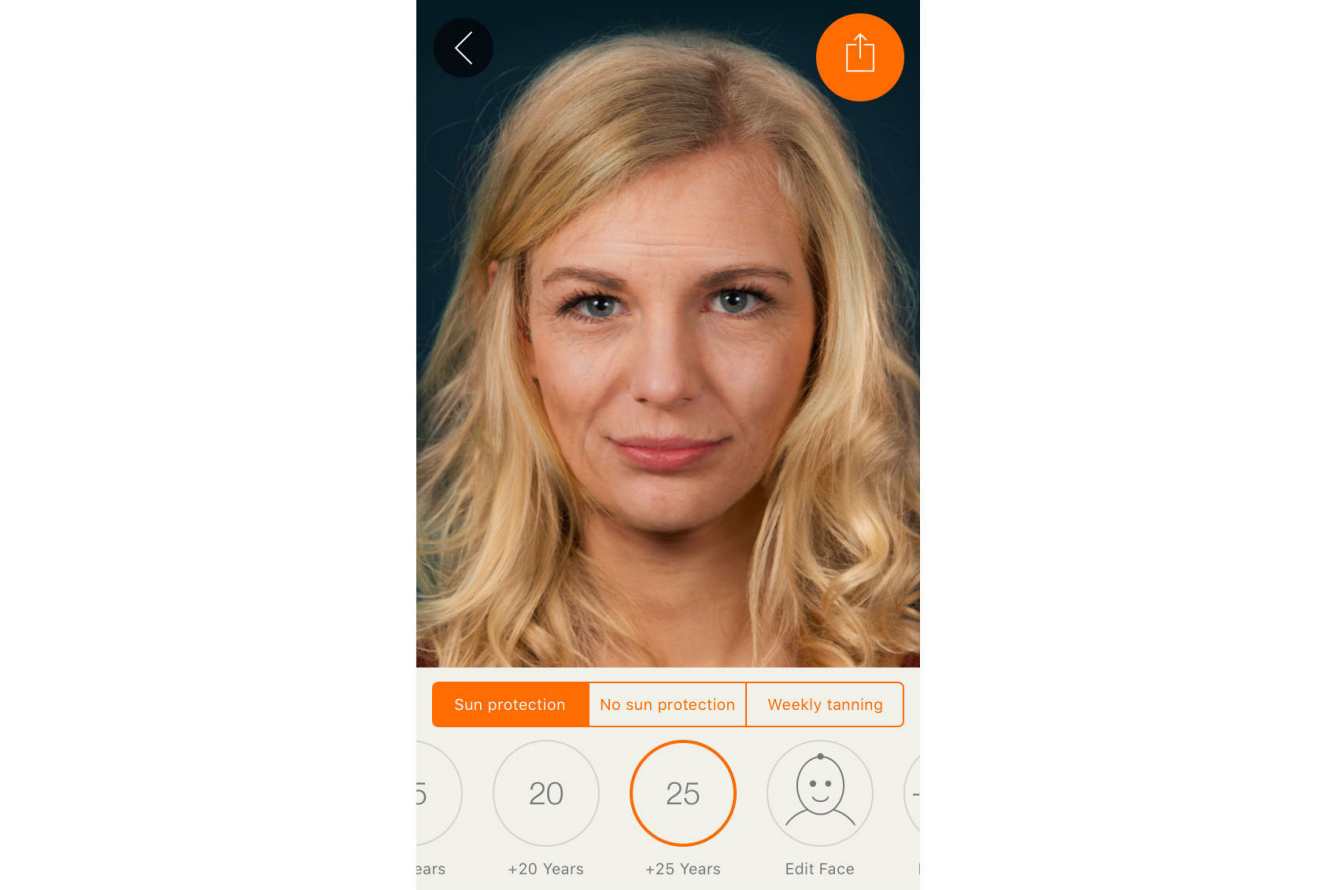
Effect view of the app: 25 years of aging with applied sun protection.

**Figure 3:**
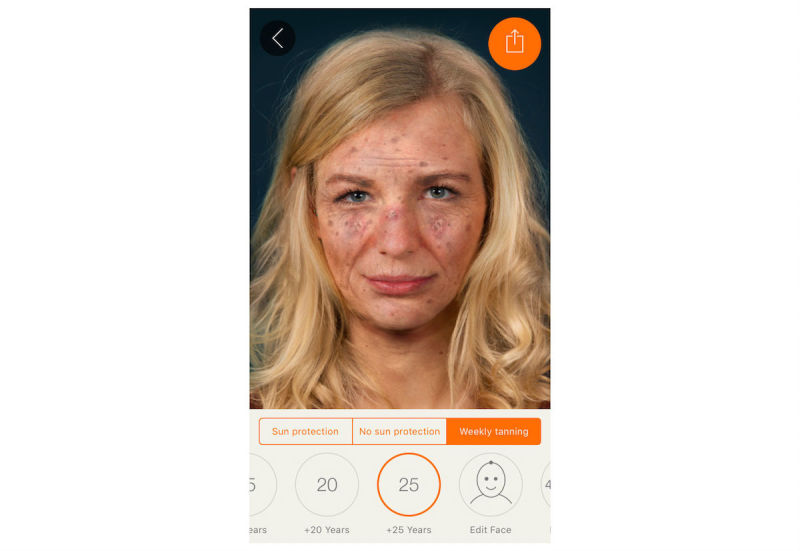
Effect view of the app: weekly tanning for 25 years (maximum effect) with a total of 3 actinic keratoses visible, multiple solar lentigines, age spots, and prominent solar elastosis.

In the first 15-minute phase, we used the displayed face of one student volunteer to show the app’s altering features to the peer group, providing an incentive for the rest of the class to test the app. In front of their peers and teachers, students could interact with their own animated face via touch (coughing, sneezing, etc) and display their future self based on their skin type (Figure 1) or use of sun protection (Figure 2) or tanning beds (Figure 3) at 5, 10, 15, 20, or 25 years in the future. Multiple device displays can be projected simultaneously, which we used to consolidate the altering measures with graphics (eg, to explain skin atrophy and solar elastosis). We implemented mirroring with a Galaxy Tab A tablet computer (Samsung, Seoul, South Korea) via Apple’s AirPlay interface (Apple Inc) using the app Mirroring360 (Splashtop Inc) for the Android operating system (Google Inc).

In the second 15-minute phase, we encouraged students to try the app on their own device or one of the tablet computers provided for students who did not own a smartphone or did not download the app. Both the provided tablet computers and the students’ own smartphones were connected to the projector.

### Data Collection

We gathered the students’ sociodemographic data (sex, age, school type) and their risk profile (skin type, sex, age, sunburn in the past, sunbed use) directly after the intervention via an anonymous survey. We captured their reactions toward the intervention via 6 items on a 5-point Likert scale (ranging from 1=strongly agree to 5=strongly disagree): (1) change of intentions (2 items: indoor vs outdoor tanning); and (2) perceived reactions of the peer group on change in attractiveness (2 items: indoor vs outdoor tanning), whether they perceived the intervention as fun (1 item), and perceived effects of the app as realistic (1 item). The items have been previously used in other published studies [30,45] and were pretested in advance in accordance with the guidelines for good epidemiologic practice [46].

### Ethics Approval

The study received ethics approval from the ethics committee at the University of Essen (17-7587-BO).

## Results

**Figure 4:**
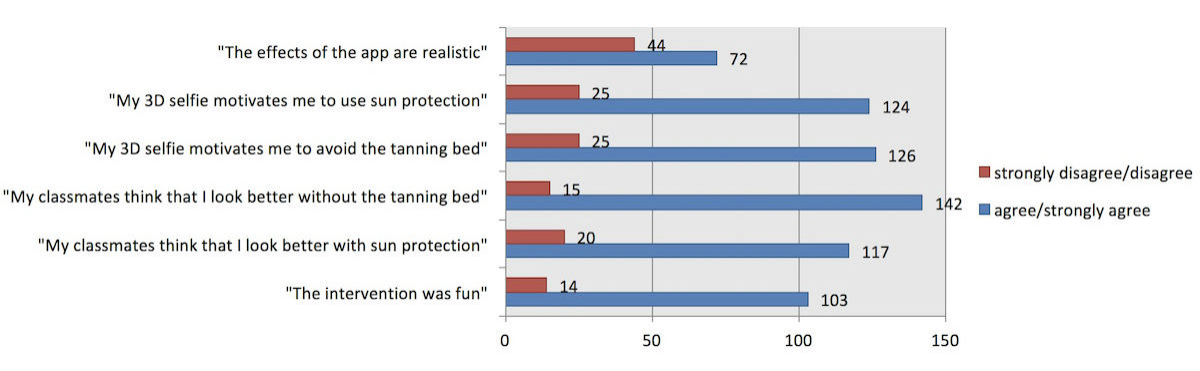
Overall results of the whole sample. 3D: 3-dimensional.

**Figure 5:**
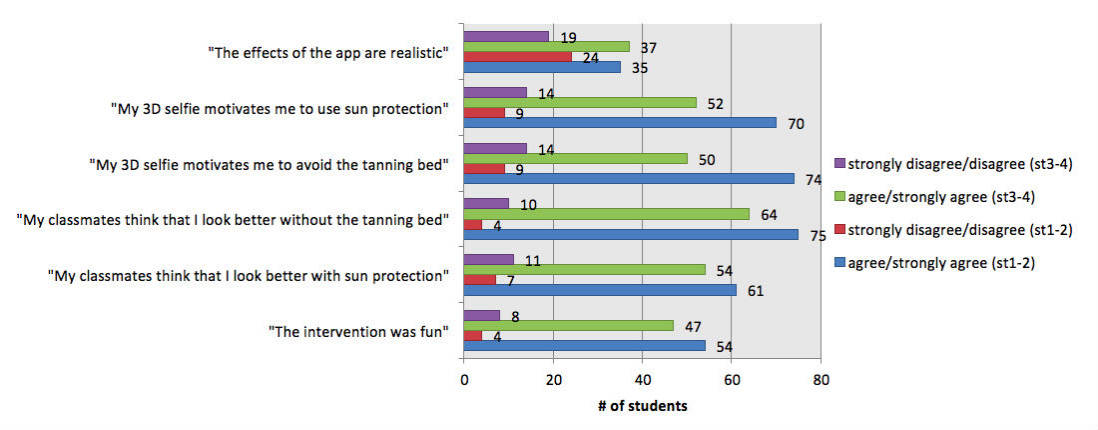
Results by Fitzpatrick skin types (st): 1-2 vs 3-6. 3D: 3-dimensional.

**Figure 6:**
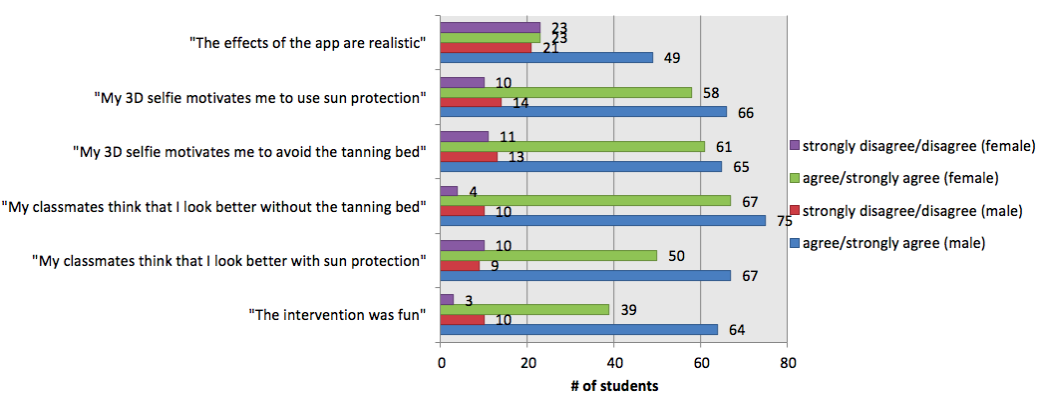
Results by sex: females vs males. 3D: 3-dimensional.

**Figure 7:**
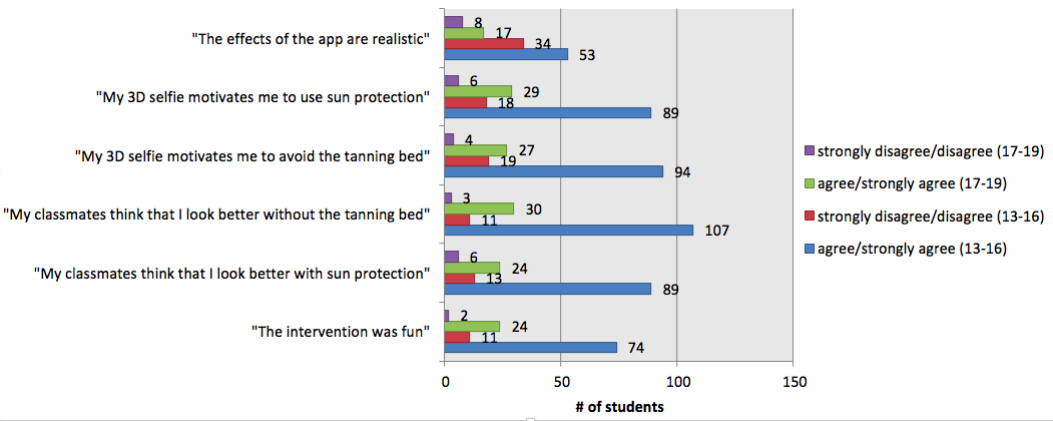
Results by age group: 13-16 years vs 17-19 years. 3D: 3-dimensional.

We analyzed all data for the group as a whole (Figure 4) but also to learn about how well the app was received by different Fitzpatrick skin types (Figure 5), sex (Figure 6), and age groups (Figure 7). All figures are based on the data provided in Multimedia Appendix 2 and give total numbers rather than percentages to adjust for the 5-point Likert scale design of the items.

### Realism of the Created Selfies

In our sample, we measured overall agreement with the subjective realism of the created selfies (72/202, 35.6% strongly agreed or agreed on realism, while n=44, 21.8% disagreed or strongly disagreed, and 42.6% (n=86) were not sure; Figure 4). These results varied notably in males (n=49, 44.5% agreement; n=21, 19.1% disagreement) versus females (n=23, 25.3% agreement; n=23, 25.3% disagreement; Figure 6) but not in other subgroups.

### Motivation to Reduce Ultraviolet Exposure

We measured more than 60% agreement in both items that measured motivation to reduce UV exposure and only 12.5% disagreement (n=126, 63.0% agreed or strongly agreed that their 3D selfie motivated them to avoid the tanning bed and n=124, 61.7% to increase use of sun protection). Only 25 (12.5%) disagreed or strongly disagreed with this item and with increased use of sun protection in our sample. The perceived effect on motivation was larger in participants with Fitzpatrick skin types 1-2 in both tanning bed avoidance (n=74, 71.8% agreement in skin types 1-2 vs n=50, 53.8% agreement in skin types 3-6) and increased use of sun protection (n=70, 68.0% agreement in skin types 1-2 vs n=52, 55.3% agreement in skin types 3-6; Figure 5), and also positively correlated with higher age (Figure 7, Multimedia Appendix 2).

### Perceived Subjective Norm During the Mirroring Intervention

The 2 items measuring the reactions of the peer group toward the individual selfie showed positive peer pressure in regard to both use of sun protection (n=117, 57.9%) and tanning bed avoidance (n=142, 72.1%; Figure 4). The subjective norm on decreasing UV exposure in order to look more attractive was perceived by a higher percentage of participants with Fitzpatrick skin types 1-2 than types 3-6, especially for tanning beds (Figure 5, Multimedia Appendix 2).

### Global Feedback

A majority of participants claimed that they perceived the intervention as fun (n=103, 51.2% agreement vs n=14, 7.0% disagreement), and the fraction agreeing that the intervention was fun was at least two times larger than the fraction disagreeing throughout all subgroups. However, this perception was notably more prevalent in male participants (n=64, 59.3%) than in female participants (n=39, 42.4%; Figure 6). A total of 79 (39.1%) of participants reported that they would try the app again later on and 57 (28.4%) planned to show the app to another person after school (Multimedia Appendix 2).

## Discussion

While multiple planned and completed trials on skin cancer prevention apps have been indexed in PubMed [47-51], to our knowledge this is the first implementation of an app-based intervention to prevent melanoma in the school setting.

Our data suggest that such an intervention is effective in changing the predictors of behavior in young risk groups and introduces a way of yielding peer-group effects in accordance with the theory of planned behavior.

### Interpretation

Available data on appearance-based behavioral change settings reveal that photoaging interventions appear to be more effective for girls [31]. In our sample, the female participants perceived the intervention as less realistic in comparison with males (n=23, 25.3% agreement vs n=23, 25.3% disagreement for females) versus males (n=49, 44.5% agreement vs n=21, 19.1% disagreement; Figure 6). However, it is notable that the relatively low perception of realism by both sexes was not accompanied by low scores in the global feedback, motivation to change behavior, or subjective norm categories. Even though the questionnaires were anonymous, which reduces confounding effects in surveys, we cannot entirely rule out that the self-reported data may have been influenced by a social desirability bias.

Previous publications stressed the relevance of targeting especially fair-skinned individuals with Fitzpatrick skin type of 1 or 2, as these are less protected against UV radiation and thus have a significantly greater risk for skin cancer [1]. In our sample, the perceived effect on motivation was larger in participants with Fitzpatrick skin types 1-2 in both tanning bed avoidance (n=74, 71.8% agreement in skin types 1-2 vs n=50, 53.8% agreement in skin types 3-6) and increased use of sun protection (n=70, 68.0% agreement in skin types 1-2 vs n=52, 55.3% agreement in skin types 3-6; Figure 5, Multimedia Appendix 2), which could be explained by the Sunface app having stronger altering effects in lighter skin than in darker skin types.

Our data also indicate that photoaging interventions have a higher impact in late adolescence (17-19 years) than in early adolescence (13-16 years), but both age groups appear to benefit (Multimedia Appendix 2). We hypothesize that this effect is due to the higher relevance of wrinkle formation (ie, due to solar elastosis) in late adolescence than in early adolescence.

### Conclusions

We present a novel way of integrating photoaging in school-based melanoma prevention, which affects the students’ peer group, considers the predictors of UV exposure in accordance with the theory of planned behavior, and is particularly effective in changing behavioral predictors in fair-skinned adolescents (Fitzpatrick skin types 1 and 2). Further research is necessary to evaluate the intervention’s prospective effects on adolescents of various cultural backgrounds.

## Appendix

Multimedia Appendix 1 Shared video of the Sunface app with 15 years of no sun protection shown on a 3D-animated selfie.

Multimedia Appendix 2 Questionnaire results.

## Abbreviations

3D: 3-dimensional
UV: ultraviolet
