# Supplementary material for: Photoaging Mobile Apps in School-Based Melanoma Prevention: Pilot Study
Source: J Med Internet Res. 2017 Sep 8;19(9):e319. doi: 10.2196/jmir.8661 (PMC5610355; doi:10.2196/jmir.8661)
Supplement: Multimedia Appendix 2 [file jmir_v19i9e319_app2.pdf]

5-point-Likert-scales (4-5= agree/strongly agree and 1-2=strongly disagree/disagree)	total (n/%)	Fitzpatrick skin type 1-2	Fitzpatrick skin type 3-6	age 13-16	age 17-19	male	female	I had a sunburn in the past	I used the tanning bed in the past year	low education level
"The intervention was fun": 4-5 / **1-2**	103/51.2% **14/7.0%**	54/52.9% **4/3.9%**	47/49.5% **8/8.4%**	74/48.7% **11/7.2%**	24/58.5% **2/4.9%**	64/59.3% **10/9.3%**	39/42.4% **3/3.3%**	77/50.3% **11/7.2%**	9/75.0% **0/0.0%**	42/60.9% **4/5.8%**
My classmates think that I look better with sun protection: 4-5/**1-2**	117/57.9% **20/9.9%**	61/59.2% **7/6.8%**	54/56.8% **11/11.6%**	89/58.2% **13/8.5%**	24/58.5% **6/14.6%**	67/60.9% **9/8.2%**	50/54.9% **10/11.0%**	94/60.6% **15/9.7%**	9/75.0% **1/8.3%**	35/50.7% **15/21.7%**
My classmates think that I look better without the tanning bed: 4-5/**1-2**	142/72.1% **15/7.6%**	75/76.5% **4/4.1%**	64/67.4% **10/10.5%**	107/71.3% **11/7.3%**	30/75.0% **3/7.5%**	75/70.8% **10/9.4%**	67/74.4% **4/4.4%**	113/74.3% **12/7.9%**	10/83.3% **1/8.3%**	54/79.4% **6/8.8%**
I want to try the app again later on: 4-5/**1-2**	79/39.1% **66/32.7%**	45/43.3% **31/29.8%**	32/34.0% **33/35.1%**	57/37.0% **54/35.1%**	19/47.5% **11/27.5%**	44/40.4% **35/32.1%**	34/37.0% **31/33.7%**	57/36.8% **51/32.9%**	7/58.3% **1/8.3%**	36/52.2% **20/29.0%**
I will show the app to other persons: 4-5 /**1-2**	57/28.4% **67/33.3%**	30/29.1% **29/28.2%**	24/25.5% **37/39.4%**	37/24.0% **60/39.0%**	17/42.5% **7/17.5%**	36/33.0% **42/38.5%**	20/22.0% **25/27.5%**	43/27.6% **57/36.5%**	6/50.0% **2/16.7%**	27/40.3% **15/22.4%**
My 3D selfie motivates me to avoid the tanning bed: 4-5/**1-2**	126/63.0% **25/12.5%**	74/71.8% **9/8.7%**	50/53.8% **14/15.1%**	94/61.8% **19/12.5%**	27/67.5% **4/10.0%**	65/59.1% **13/11.8%**	61/68.5% **11/12.4%**	99/64.7% **19/12.4%**	9/75.0% **1/8.3%**	44/64.7% **9/13.2%**
My 3D selfie motivates me to use sun protection: 4-5/**1-2**	124/61.7% **25/12.5%**	70/68.0% **9/8.7%**	52/55.3% **14/14.9%**	89/58.2% **18/11.8%**	29/72.5% **6/15.0%**	66/60.0% **14/12.7%**	58/64.4% **10/11.1%**	96/62.3% **19/12.3%**	10/83.3% **1/8.3%**	49/72.1% **8/11.8%**
The effects of the app are realistic: 4-5/**1-2**	72/35.6% **44/21.8%**	35/34.0% **24/23.3%**	37/38.9% **19/20.0%**	53/34.6% **34/22.2%**	17/41.5% **8/19.5%**	49/44.5% **21/19.1%**	23/25.3% **23/25.3%**	57/37.0% **34/22.1%**	8/66.7% **1/8.3%**	24/34.8% **15/21.7%**
I had a sunburn in the past (yes)	157/77.0%	86/82.7%	67/69.8%	126/81.3%	24/58.5%	87/79.1%	69/74.2%	157/100.0 %	10/83.8%	40/58.8%
I used the tanning bed in the past year (yes)	12/5.9%	8/7.7%	4/4.1%	7/4.5%	5/12.2%	4/3.6%	8/8.6%	10/6.4%	12/100.0 %	8/11.6%
I own a smartphone (yes)	201/98.5%	104/100%	94/97.9%	155/ 99.4%	39/97.5%	110/100%	91/97.8%	155/98.7%	10/83.3%	66/97.1%
